# Auto-Selection of an Optimal Sparse Matrix Format in the Neuro-Simulator ANNarchy

**DOI:** 10.3389/fninf.2022.877945

**Published:** 2022-05-23

**Authors:** Helge Ülo Dinkelbach, Badr-Eddine Bouhlal, Julien Vitay, Fred H. Hamker

**Affiliations:** Department of Computer Science, Chemnitz University of Technology, Chemnitz, Germany

**Keywords:** neural simulator, rate-coded networks, auto-tuning, code generation, CUDA

## Abstract

Modern neuro-simulators provide efficient implementations of simulation kernels on various parallel hardware (multi-core CPUs, distributed CPUs, GPUs), thereby supporting the simulation of increasingly large and complex biologically realistic networks. However, the optimal configuration of the parallel hardware and computational kernels depends on the exact structure of the network to be simulated. For example, the computation time of rate-coded neural networks is generally limited by the available memory bandwidth, and consequently, the organization of the data in memory will strongly influence the performance for different connectivity matrices. We pinpoint the role of sparse matrix formats implemented in the neuro-simulator ANNarchy with respect to computation time. Rather than asking the user to identify the best data structures required for a given network and platform, such a decision could also be carried out by the neuro-simulator. However, it requires heuristics that need to be adapted over time for the available hardware. The present study investigates how machine learning methods can be used to identify appropriate implementations for a specific network. We employ an artificial neural network to develop a predictive model to help the developer select the optimal sparse matrix format. The model is first trained offline using a set of training examples on a particular hardware platform. The learned model can then predict the execution time of different matrix formats and decide on the best option for a specific network. Our experimental results show that using up to 3,000 examples of random network configurations (i.e., different population sizes as well as variable connectivity), our approach effectively selects the appropriate configuration, providing over 93% accuracy in predicting the suitable format on three different NVIDIA devices.

## 1. Introduction

Models in computational neuroscience are implemented with different degrees of biological detail. Particularly at the systems-level, a significant subset of models incorporate dynamic rate-coded neurons to explain emergent functions of such networks and link them to experimental data. In such networks, neurons are connected to other neurons by axons and synapses, whose joint effect is captured by so-called weights *w*_*ij*_ and describes in how far the firing rate *x*_*i*_ of a presynaptic neuron *i* affects the firing of a post-synaptic neuron *j*. As outlined by Dinkelbach et al. (2012), the sum of weighted inputs *w*_*ij*_·*x*_*i*_, required to be computed at each time step, is the dominating operation in large-scale rate-coded neural networks, well before other operations such as the numerical integration of ordinary differential equations (ODE). It was shown using a simplified network model that the choice of either a multi-core CPU or a GPU (Graphical Processing Unit) as the computing backend depends on the network's structure. GPU implementations were more efficient on mid- and large-scale networks in comparison to a multi-core CPU implementation. Dinkelbach et al. (2019) observed for a linear rate-coded model that the network had to consist of thousands of neurons in order to utilize a GPU effectively.

When applied on populations of neurons, the weighted sum of synaptic inputs can be computed by a sparse matrix-vector multiplication (SpMV) between a (sparse) matrix **W** and a dense vector $\vec{x}$ which results in a dense vector $\vec{y}$:


(1) $$\begin{array}{l} \vec{y}=\text{W}\times \vec{x}. \end{array}$$

The SpMV operation, which is a central kernel in many scientific applications, is considered to be memory-bound and is impaired by irregular access patterns to the dense vector $\vec{x}$ (e.g., Temam and Jalby, 1992; Goumas et al., 2008; Williams et al., 2009; Greathouse and Daga, 2014; Langr and Tvrdik, 2016; Filippone et al., 2017). While each non-zero element of **W** is only accessed once in the SpMV operation, there is frequent access to $\vec{x}$ at different positions (e.g., Williams et al., 2009). Depending on the distribution of the non-zeros within a row of the matrix, this can lead to cache misses or re-loads, leading to noticeable performance decreases on CPUs and especially on GPUs (e.g., shown in Dinkelbach et al., 2012). For optimal performance, the number of these scattered accesses should be reduced, for example through a reuse, efficient caching (CPU-oriented architectures) or pre-loading into shared memory (GPU) of the dense vector (e.g., Goumas et al., 2008; Williams et al., 2009; Greathouse and Daga, 2014). To overcome this issue, many different formats were proposed to perform the SpMV operation efficiently on single-core, multi-core CPUs or GPUs (see Langr and Tvrdik, 2016; Filippone et al., 2017 for more details). Nevertheless, understanding the efficiency of applied optimizations can be difficult as the interaction of optimizations with each other or the underlying hardware is hard to predict (see Goumas et al., 2008; Balaprakash et al., 2018 for a detailed discussion). The efficiency of a single optimization may depend on the matrix as well as on the specific platform as demonstrated in the work of Williams et al. (2009). However, the efficiency of an implementation can also change by advancements made by compilers and hardware as pointed out by Steinberger et al. (2016).

Due to the generally unknown sparsity of a matrix, choosing an efficient parallel implementation of the SpMV operation for a given matrix is therefore an important and hard problem (e.g., Liu and Vinter, 2015b; Lehnert et al., 2016; Hou et al., 2017). However, there exists some knowledge about which given format is more suitable for a given matrix. For example, Vázquez et al. (2011) and Sedaghati et al. (2015) suggest that the density of a matrix is a guiding factor for the selection of a particular data structure. Furthermore, as shown by Vázquez et al. (2011), the variability of row lengths can be a relevant criterion in the selection of data formats.

Machine learning methods received increasing attention for the tuning of implementations at various levels, including the selection of code variants, parallelization strategies, or even complete algorithms (see Balaprakash et al., 2018 for a recent review). Modern multi-core CPUs and GPUs in combination with compilers offer a rich possibility for programmers to adapt their code to increase performance. Therefore, the possible search space even for relatively simple operations can reach millions of configurations (e.g., as shown by Datta et al., 2008; Ganapathi et al., 2009 for the stencil operation). Auto-tuning methods considering the SpMV operation were investigated for single-thread, multi-core as well as GPU configurations either using hand-tuning (e.g., Choi et al., 2010), heuristics (e.g., Whaley et al., 2001; Sedaghati et al., 2015), or machine learning methods (e.g., Ganapathi et al., 2009; Benatia et al., 2018; Pichel and Pateiro-Lopez, 2018; Chen et al., 2019). As hardware and algorithms steadily evolve, it is important to integrate auto-tuning principles inside the specific application. Such an integration allows to adjust the build process considering the target platform (Balaprakash et al., 2018).

The present article shows that implementing different sparse matrix formats in a neural simulator can improve the overall performance of rate-coded neural networks. We present a two-stage heuristic already embedded in our neural simulation framework ANNarchy (Artificial Neural Networks architect, Vitay et al., 2015). We also demonstrate that the performance can be improved by integrating machine learning methods. This should help developers of neural network models selecting a suitable data structure representation for their specific network.

## 2. Related Work

### 2.1. Sparse Matrix Formats for SpMV

As outlined in the introduction, the SpMV operation has been thoroughly investigated and several sparse matrix formats have been proposed. The following collection of formats is just a short overview and by no means exhaustive. For more details, refer to the reviews of Langr and Tvrdik (2016) and Filippone et al. (2017).

**Figure 1:**
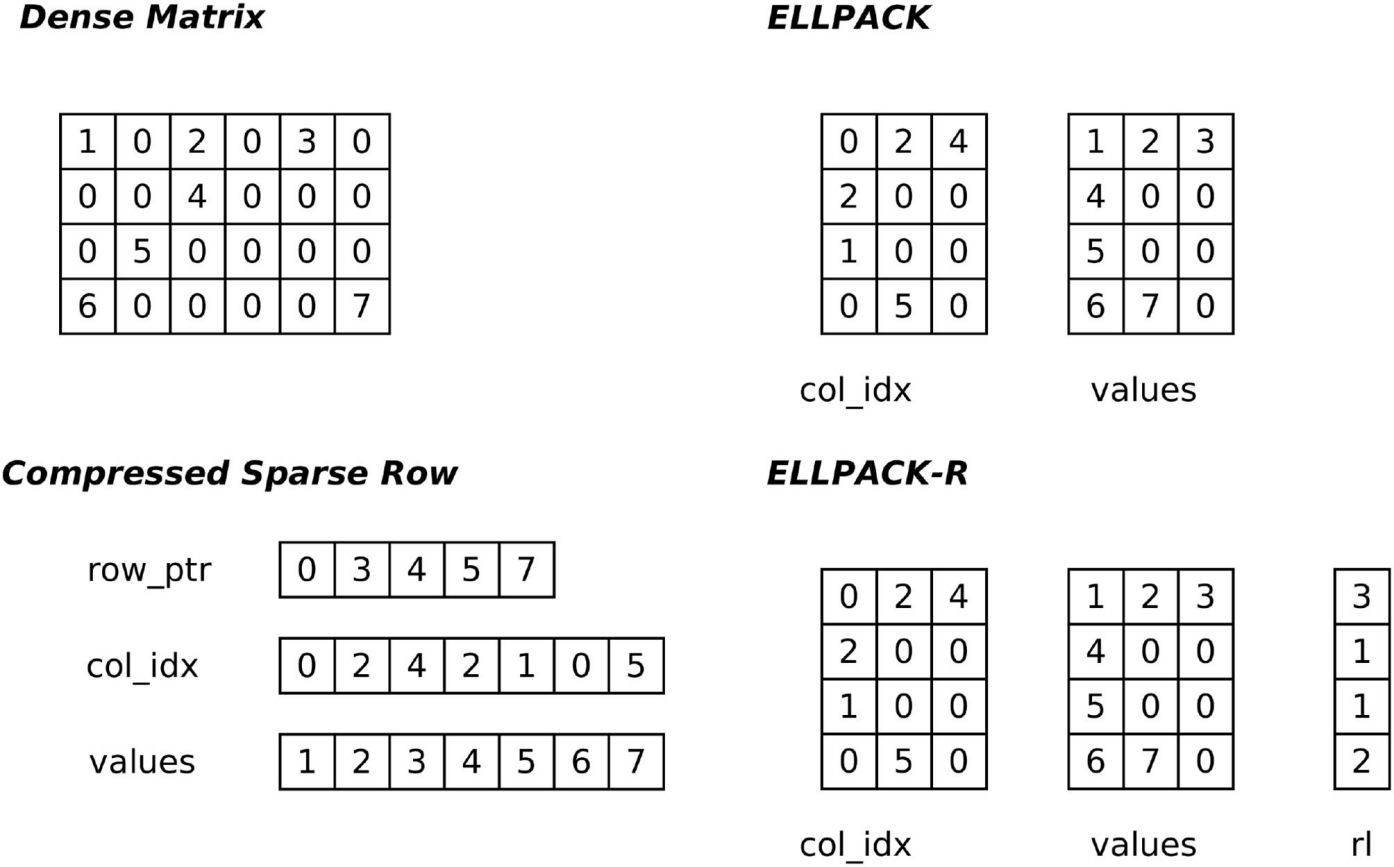
Schematic representation of the compressed sparse row (CSR), ELLPACK, and ELLPACK-R formats derived from a dense matrix. The CSR format comprises three dense vectors: a *row_ptr* array where the begin and end of subsequent rows are encoded. These indices are needed to select the correct values from the column index and value array. Contrary to CSR, in ELLPACK/ELLPACK-R the column indices and the values are encoded in dense matrices. The ELLPACK-R has an additional row-length (rl) array to encode the row lengths to spare the index checking.

Probably the most common and well-known format is the compressed sparse row (or Yale) format (CSR). The non-zeros of each row are stored in two arrays (one for the column indices and the other one for the values). The start and stop indices of a row are stored in a row pointer array. The ELLPACK/ITPACK format (Kincaid et al., 1989) was intended to be efficient for vector processors. This format decomposes the non-zeros into two dense matrices whose dimensions are number of rows times the maximum number of non-zeros within a row, one matrix representing the column indices, the other the values. If the matrix has heterogeneous row lengths, non-existing entries need to be marked by a neutral element, which likely creates a large memory overhead. This format is considered as GPU-friendly if the dense matrices are stored in column-major^1^ order (Bell and Garland, 2009; Vázquez et al., 2011). Vázquez et al. (2011) proposed an extended version, ELLPACK-R, which introduces an additional row-length array to encode varying row lengths instead of checking each matrix entry with an if-clause. An overview of the different sparse matrix formats is depicted in Figure 1.

### 2.2. ANNarchy

The ANNarchy neural simulator is written in Python and intended for the simulation of biologically detailed neural networks. The equation-based interface of ANNarchy allows a flexible and easy definition of the neuron and synapse models (Vitay et al., 2015). Using an automatic code generation approach, the model description is transformed into C++ code allowing the use of parallel programming frameworks such as OpenMP for multi-core CPUs or CUDA for GPUs for the efficient implementation of rate-coded and spiking models (Vitay et al., 2015; Dinkelbach et al., 2019).

The current version 4.7.1.1 of ANNarchy provides several sparse matrix formats for the computation of rate-coded neural network models. In addition to the already existing list-in-list/compressed sparse row implementation (as described in Dinkelbach et al., 2012), an ELLPACK/ITPACK (Kincaid et al., 1989; Vázquez et al., 2011) and a dense matrix format have been added, which will be evaluated in Section 4. ANNarchy also implements a Hybrid format as described by Bell and Garland (2009) and a blocked sparse row (BSR) format as described by Verschoor and Jalba (2012) and Eberhardt and Hoemmen (2016), but preliminary tests have shown that those formats are not performing well in comparison to the others on the dataset used in this work, so they are omitted for the present article. We hypothesize that the structure of the matrices in our dataset, i.e., a relatively homogeneous row length (for Hybrid) and a high scattering across the matrix (for BSR), are limiting factors for these data formats.

Further, we extended our code generation approach to allow auto-vectorization (using compiler hints e.g., #pragma simd) for the continuous neural and synaptic state updates by reordering the code to reduce the number of branches. We introduce for continuous transmission an implementation using AVX-512, AVX and SSE4.2 instructions^2^ to address most of the currently available CPU architectures.

### 2.3. Auto-Tuning Methods

As outlined by Balaprakash et al. (2018), auto-tuning in high-performance computing is utilized at various levels within an application. Many of these works/ideas are conjuncted with highly optimized libraries like ATLAS^3^ (Whaley et al., 2001), SPARSITY (Im et al., 2004), or OSKI (Vuduc et al., 2005). These frameworks are often not limited to the SpMV operation but implement a set of operations from the basic linear algebra (BLAS) routines. This is in contrast to optimized libraries such as clSpMV (Su and Keutzer, 2012) or SMAT (Li et al., 2013) which only focus on the SpMV operation. From our perspective, there are two types of approaches that are of special interest.

First, hand-tuning of a specific format is probably the most common approach, where data structures are adapted to the algorithm or processed data. Some examples are the CSR-like (Hou et al., 2017), ELLR-T (Vázquez et al., 2012), BCSR (Choi et al., 2010), BELLPACK (Choi et al., 2010), and sliced ELLPACK Kreutzer et al. (2014) data structures. Especially for GPUs arise the question of load balancing, i.e., how many threads should be used and how many blocks should be used for computation at the same time. The effect of the block size can already vary noticeably on a single example as demonstrated by Eberhardt and Hoemmen (2016). The performance was most consistent on a Sandy Bridge CPU in comparison to a GPU and a Xeon Phi. Guo and Wang (2010) proposed a model-driven approach for the fine-tuning of the blocked CSR and blocked ELLPACK format to tackle this issue.

The second class of approaches is the selection of a suitable format for a given matrix, as investigated by Li et al. (2013), Greathouse and Daga (2014), Sedaghati et al. (2015), or Benatia et al. (2018). The main idea is to derive the decision based on a set of features. The mapping of features into a decision can be based on either heuristics or machine learning methods. For instance, Lehnert et al. (2016) have shown that performance prediction using machine learning methods can outperform explicit performance models. The predicted computation time is then used to derive the matrix format decision. In the present manuscript we will follow the second class of approaches, more precisely the work of Lehnert et al. (2016) and Benatia et al. (2018), using regression techniques to predict the performance of a sparse matrix format applied on a given matrix.

## 3. Methods

Our focus is to develop an efficient tool that can predict with high accuracy the suitable format for each connectivity matrix of a specific neural network. In the following, we propose two methods for matrix format selection: The first is based on a simple heuristic (Section 3.1) and the second uses a machine learning model (Section 3.2) for predicting the appropriate format.

### 3.1. Two-Stage Heuristic for Format Selection on GPUs

We followed the idea of Sedaghati et al. (2015), who analyzed the obtained GFLOPS (floating operations per second, see Section 4 for a more detailed description) on several matrices for potential correlations. In their work, they showed that a quite good heuristic can be based on the fraction of non-zeros. We are going to compare three available implementations: the CSR format using an updated version of the algorithm presented in Dinkelbach et al. (2012), the ELLPACK-R presented in Vázquez et al. (2011) as well as a dense matrix representation.

There are several factors influencing the performance achieved with a given implementation on GPUs. One crucial fact is to ensure coalesced memory access toward accessed data (e.g., Bell and Garland, 2009; Dinkelbach et al., 2012; Yavuz et al., 2016). A memory access is considered as coalesced if all threads within a half-warp^4^ can use the data loaded from a 32-, 64-, or 128-byte segment (Bell and Garland, 2009). One key difference between the implementations of the SpMV using CSR and ELLPACK-R is that they are parallelized over different dimensions: while our CSR implementation computes one row per warp, a warp in ELLPACK-R computes a set of rows at the same time.

**Figure 2:**
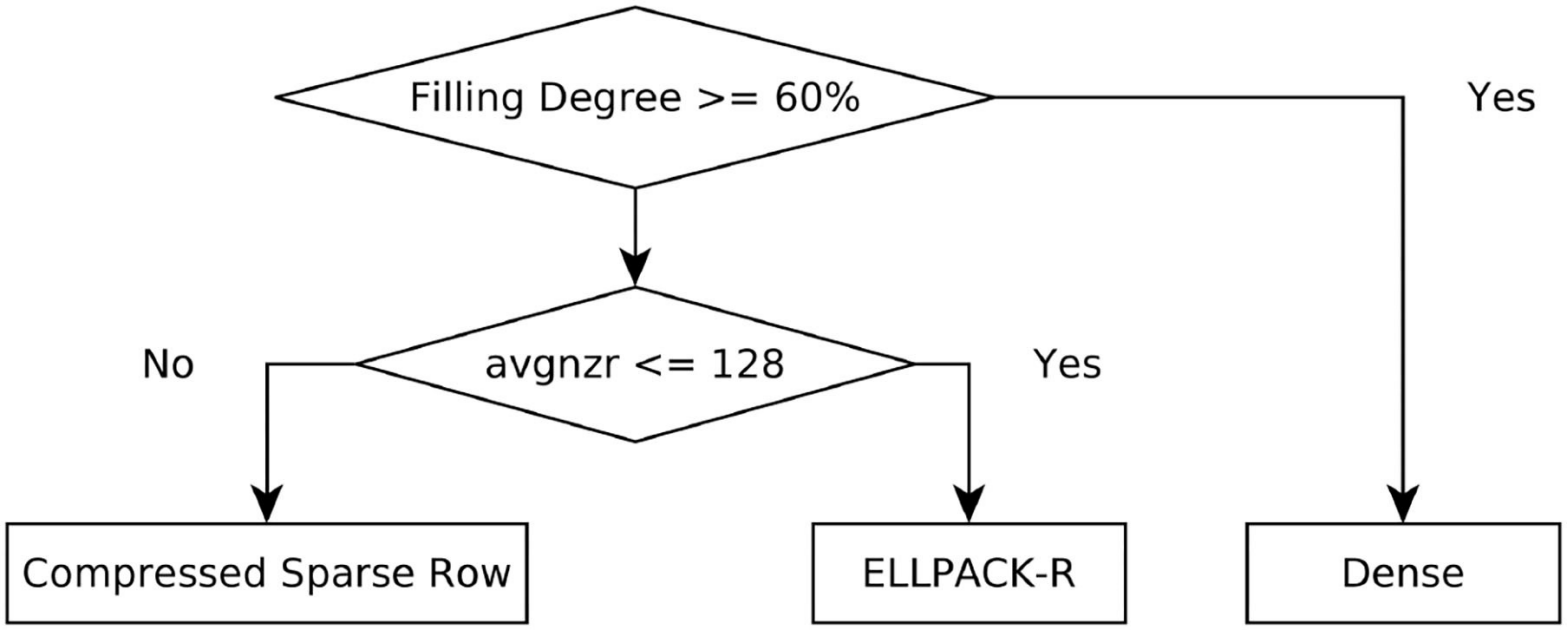
Two-stage heuristic for the matrix format selection on GPUs. The threshold values for both decision points were selected based on the analysis of our datasets (see Supplementary Material, Section 3 for more details).

Considering these different computation patterns and the fact that a dense matrix is efficient for densely packed matrices, one can obtain a simple decision tree as depicted in Figure 2. The decision is two-fold: first we decide based on the matrix density, i.e., the ratio of nonzeros to the total number of elements in the matrix, whether the density is greater than a threshold. The matrix is considered as dense in this case. Otherwise the average number of non-zeros in a row (avgnzr) is considered. If this value is lower or equal to 128, the ELLPACK-R format is selected, otherwise CSR is chosen. The threshold for the first decision stage is derived from observations made on the experiments shown in Section 4.1. However, these observations should be verified if they generalize, therefore we also analyzed the 3,000 data points generated for the machine learning model (as shown in Supplementary Material, Section 3) and confirmed that the threshold of 60% is appropriate for this decision stage. The threshold for the second decision stage is based on theoretical knowledge about the computation patterns. The threshold should be chosen as a multiple of the warp size to ensure a full utilization of the computation blocks. We analyzed the performance as a function of the average number of non-zeros in a row (see Supplementary Material, Section 3) and derived the value of 128 as suitable decision threshold for our dataset. However, the analysis also suggests that this threshold could be fine-tuned to a specific CUDA device to achieve an optimal performance of the heuristic.

### 3.2. Format Selection Using Machine Learning

The heuristic approach is limited, as it is difficult to identify differences arising from the execution of a given implementation on different devices (see Section 4.2). To be efficient on diverse devices, one would need to fine-tune the decision parameters for each device. Therefore, it would be useful to have an automatic selection which can be adapted through machine learning to data obtained from each device.

The implementation of the prediction model requires three general steps. The first step is made offline and consists in generating the dataset necessary for the training and testing of the model. The second step is also offline and consists in training the model and testing it. The last one is online and consists of using the model and performing predictions that help in selecting the most suitable format.

#### 3.2.1. Creation of the Dataset

For our benchmark, we follow a scheme similar to Dinkelbach et al. (2012). We create two populations in ANNarchy. The population sizes were randomly chosen from a fixed set of sizes within the range of 1,000–20,000 neurons. We create a projection between those two populations, which will be referred to as the connectivity matrix in this section. For the creation of this matrix, we either use a random probability (in the range of 1–100%) or a fixed number of entries per row (ranging from 128–4,096 entries). Using this scheme, we create 3,000 different network configurations. Each network is then generated, compiled and simulated for 1,000 steps using each data structure (in this case the CSR, ELLPACK-R and dense matrix formats). At the end of this procedure, we obtained 3,000 data points which consist of a list of features (described in the next section), the achieved computational time for each of the three formats and the format which would be chosen by the heuristic.

#### 3.2.2. Feature Selection

The computation time of a rate-coded network heavily depends on the number of connections between the different populations. Since these various connections are structured in the format of sparse matrices, we focus on the properties of this particular type of matrix to define the relevant input features to the auto-tuning network. We derive for the matrices the features depicted in Table 1.

**Table 1:** A set of features used to characterize the sparse matrices.

**Features**	**Description**
*N*	Number of rows in the matrix
M	Number of columns in the matrix
NNZ	Number of nonzeros in the matrix
DES	Density of the matrix
AVGNZR	Average number of nonzeros per row
MINNZR	Minimum number of nonzeros per row
MAXNZR	Maximum number of nonzeros per row

This set of features is a subset of features which are typically used in the SpMV auto-tuning literature (e.g., Li et al., 2013; Lehnert et al., 2016; Benatia et al., 2018; Chen et al., 2019). In particular, the work of Lehnert et al. (2016) and Benatia et al. (2018) suggests that the set of features used to detect a format depends on the format itself. For instance, we left out the difference between the maximum number of nonzeros (MAXNZR) and the average nonzeros per row (AVGNZR) as our preliminary experiments indicated that this feature is not helpful on our dataset. Considering the work of Vázquez et al. (2011) and Benatia et al. (2018), we believe this feature is a helpful indicator for the Hybrid format which is not used in the present work (see Section 5 for more details) and thus we omit this criterion. Li et al. (2013) proposed two additional values to characterize diagonals in matrices which might indicate the usage of diagonal formats. It is worth noting that not all approaches use such features. Pichel and Pateiro-Lopez (2018) use for example, an image-like tensor to represent the features of the connectivity matrix which is scaled down to be used as input to a convolutional neural network (AlexNet, Krizhevsky et al., 2012) to derive the optimal matrix format.

#### 3.2.3. Machine Learning Model

The machine learning model is implemented using the TensorFlow (Abadi et al., 2016) library version 2.6.2^5^. The fully-connected feedforward neural network consists of an input layer with seven neurons representing the features (as discussed in Section 3.2.2), a feature normalization layer, a number of hidden layers and one output layer with three neurons. Each of these neurons represents a possible data structure: CSR, ELLPACK-R, and dense. The output of these neurons, i.e., the predicted performance for a given network in GFLOPs, is then read out to determine the fastest configuration. The hidden layers consist of rectified linear units (ReLu) and the number of layers as well as the number of units in each layer is determined by Optuna (Akiba et al., 2019), a Bayesian optimization library for hyper-parameter optimization used in many machine learning workflows. The search space is here the set of possible configurations, in our case the number of layers from 2 to 5 (motivated by the work of Benatia et al. (2018) who identified four layers as optimal for ELLPACK and five as optimal for CSR on their dataset), the number of neurons in each layer (64–256) and the learning rate (1e-7 to 1e-2). The objective function provided to Optuna is the test accuracy, an average resulting from a 5-fold cross-validation (see Section 4.4.1 for more details) without repetitions. We configured Optuna to perform 150 trials for each of the three datasets (i.e., the three CUDA devices considered in this work) and the obtained best configurations are depicted in Table 2.

**Table 2:** Best network configurations found by the Optuna library within 150 trials for each dataset.

	**NVIDIA K20m**	**NVIDIA RTX 2060**	**NVIDIA RTX 3080**
Normalization	7	7	7
Dense	119	124	155
Dense	187	195	86
Dense	199	105	85
Dense	96	127	150
Dense	/	/	66
Output	3	3	3

The optimizer is Adam with the default parameters and the learning rate is determined by Optuna. The loss function is the mean squared error (mse), as this is a regression problem.

## 4. Results

All the experiments were performed using the ANNarchy 4.7.1.1 release^6^. The measured computation times are recorded with the Python time package. When we analyze the performance in this section, we evaluate the execution of 1,000 steps within the ANNarchy neural simulator. As the populations are not defined by means of equations, the simulation time is almost equal to the execution time of the SpMV. We use in this article FLOPS (floating operations per second) as a metric to evaluate the performance, which is used commonly across the SpMV literature. This value is computed for a given data structure based on the measured computation time *t* in seconds for the 1,000 iterations (as mentioned in Section 3.2.1) and the number of nonzeros (*nnz*) in the matrix:


(2) $$\begin{array}{l} \text{FLOPS}=\frac{2\times 1,000\times nnz}{t} \end{array}$$

The factor 2 comes from the fact that the SpMV requires one multiplication and one addition for each non-zero value. For an easier handling of the values, we transform then FLOPs to GFLOPs (giga-FLOPs). Langr and Tvrdik (2016) suggest to choose compiler flags for performance comparisons in order to achieve the best possible performance. The ANNarchy framework was therefore configured to use the optimization flags *-march=native*^7^ *-O3*^8^ *-ffast-math*^9^ for the g++ compiler to enable typical optimizations. The CUDA compiler is configured without further compiler flags as *-O3* is automatically enabled for device codes^10^. For a more detailed discussion on the effect of *-ffast-math* and the CUDA compiler counterpart –*use_fast_math* we would like to refer to Supplementary Material, Section 4. The following sections will compare the performance achieved on three NVIDIA devices: a K20m, a RTX 2060, and a RTX 3080. Some hardware characteristics are provided in the Supplementary Material, Section 1.

### 4.1. Dense vs. Sparse Matrix Formats

**Figure 3:**
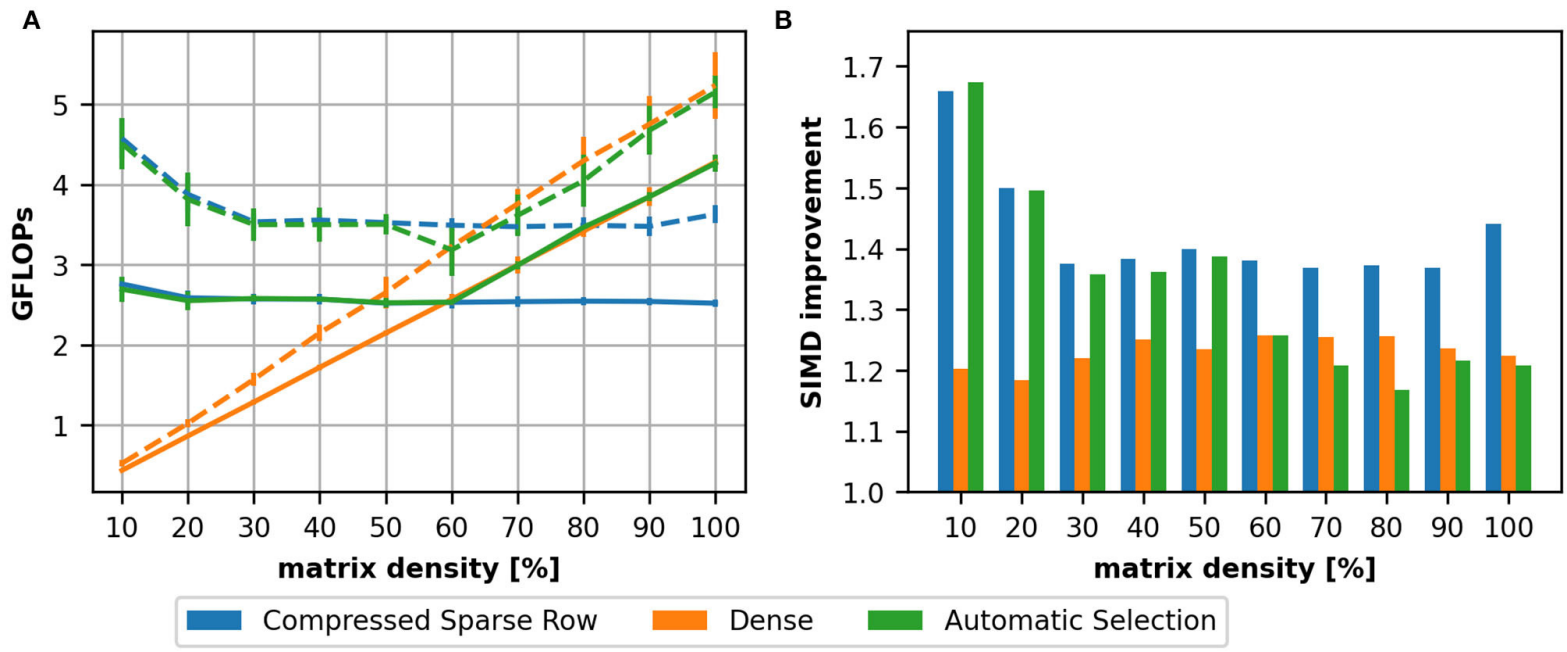
Comparison between a dense matrix representation and the compressed sparse row format on a AMD Ryzen 7 2700X using a single thread. We depict the achieved performance in GFLOPs as a function of matrix density **(A)**. In this setup we compare a 2,000 × 2,000 matrix with varying density levels and compare a CSR (blue) and dense (orange) implementation. We compared additionally the improvement by a hand-written AVX implementation (dashed line). The gained improvement by this implementation is depicted in **(B)**.

**Figure 4:**
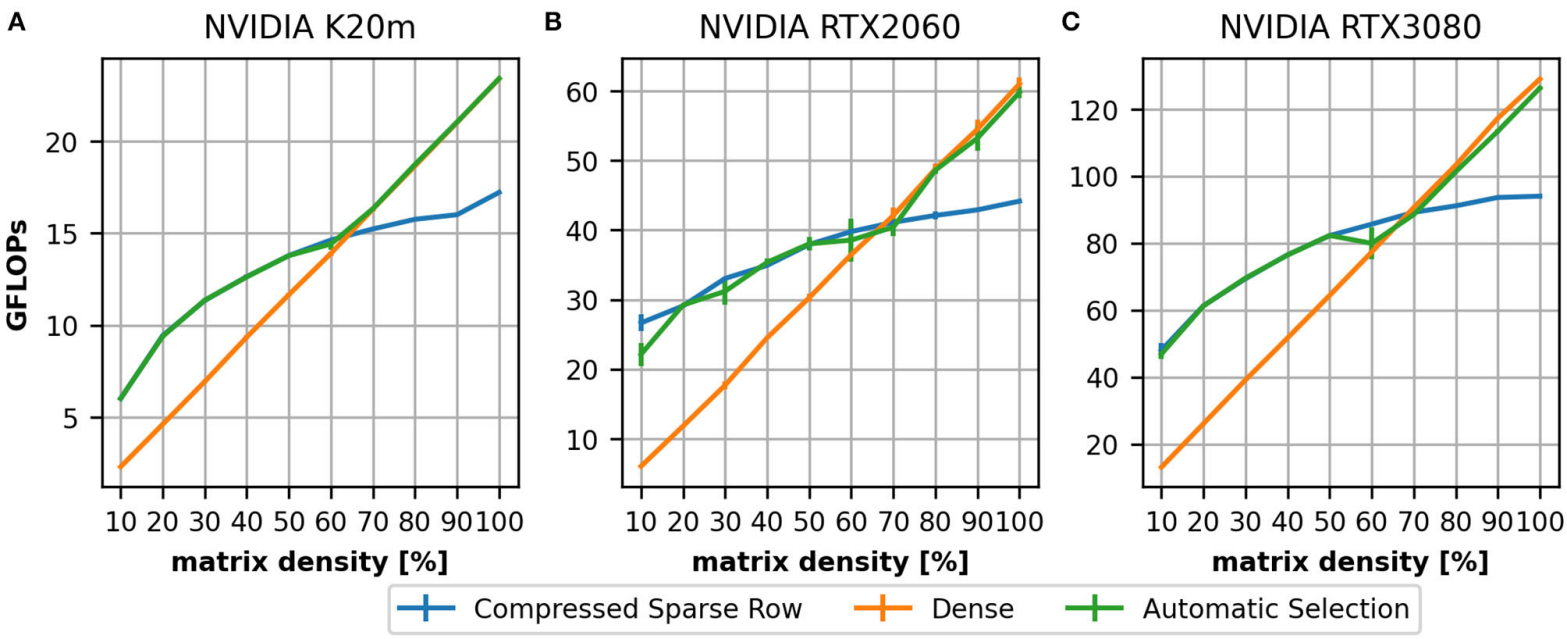
Achieved performance in GFLOPs on three devices: a NVIDIA K20m **(A)**, a NVIDIA RTX 2060 **(B)**, and a NVIDIA RTX 3080 **(C)**. As for the single thread CPU (Figure 3) we compare a CSR (blue) and dense (orange) implementation on a 2,000 times 2,000 matrix with varying filling degree. In the range of 60–70% the dense matrix representation outperforms the CSR which motivated the first stage of our heuristic.

Sparse matrix representations require a memory overhead to index the elements of a matrix (e.g., row pointers). When the matrix becomes denser, it may become inefficient to use a sparse matrix representation instead of a dense one (see Supplementary Material, Section 2 for more details). To illustrate this, we define a 2,000 × 2,000 matrix with varying sparsity levels ranging from 10% to fully-connected. We compare the achieved throughput in GFLOPs averaged across 15 runs for a single thread on a AMD Ryzen 7 2700X CPU (Figure 3) and three different NVIDIA devices (Figure 4). The CSR data structure (blue), the dense format (orange) and a format selected by the heuristic (green) are compared.

For the CPU (Figure 3A), we can see that the GFLOPs are almost constant for the CSR format, i.e., the computation time increases linearly with the number of non-zeros in the matrix, while the contrary applies for the dense matrix as the computation time is not dependent on the number of non-zeros: the achieved GFLOPs are low for sparse matrices and increase with the matrix density. As outlined in Section 2.2, we added also hand-written vectorization using AVX on the AMD Ryzen7 CPU. The results for the vectorized implementations are depicted in Figure 3A as dashed lines. The relative improvement provided by the vectorization is also depicted as a bar graph in Figure 3B. We can see that the improvement is below the theoretical maximum which would be four for double precision on an AVX-capable CPU. The reduced efficiency, especially for the dense matrix format, should be linked to the fact that the SpMV is a memory-bound problem. We also see that the improvement is almost the same for a density around 20% while the improvement achieved on the CSR depends on the density: for small densities, the implementation benefits mostly for small row lengths and the reduced memory consumption.

To evaluate the performance on GPUs we compare the K20m (Figure 4A), the RTX 2060 (Figure 4B) and the RTX 3080 (Figure 4C). On all three devices, we can see that for small densities the achieved throughput of the CSR (blue line) implementation is lower than for higher densities. This is a consequence of the implementation [as discussed in Section 3.1; more details can be found in Dinkelbach et al. (2012) for our version of the CSR and in Vázquez et al. (2011) for the ELLPACK-R format] as the thread groups processes rows together: there must be a sufficient number of elements in a row to achieve a high throughput.

In both experiments, we can see that, for higher matrix densities, the CSR format is outperformed by the dense matrix format (orange line). This motivated the first stage of our heuristic (green line). The value 60% was originally obtained on the K20m GPU. A comparison to the newer devices would suggest 70%. We have analyzed this for all examples in our dataset and determined 60% as a suitable value (see Supplementary Material, Section 3).

### 4.2. Different Sparse Matrix Formats

This section illustrates the necessity for different sparse matrix formats. We investigate the performance improvement of an ELLPACK-R and dense implementation against the CSR on three GPUs which is a criterion suggested by Langr and Tvrdik (2016). To compare the formats, we compute the ratio between the GFLOPS required by CSR and the GFLOPS of the other format. A more detailed analysis of these values is depicted in the Supplementary Material, Section 3.

**Figure 5:**
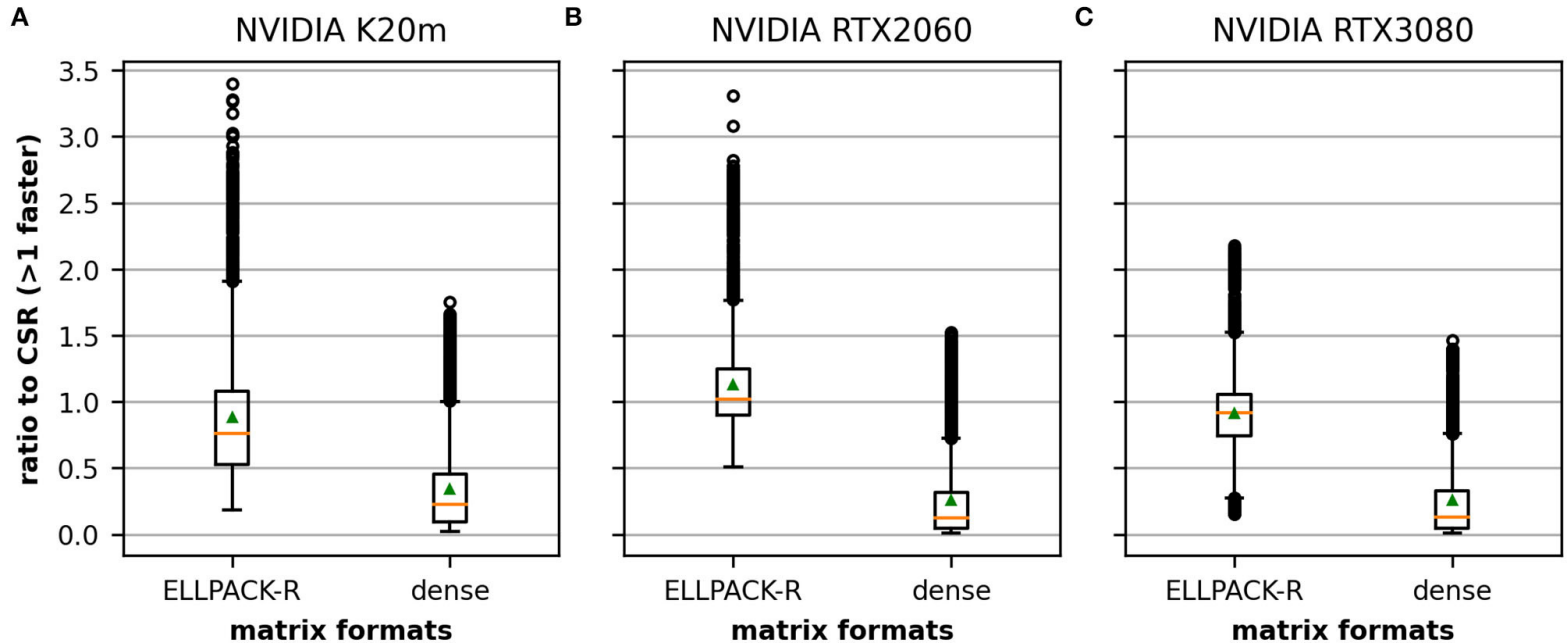
Relative performance of ELLPACK-R and dense matrices in comparison to a CSR averaged across the 3,000 matrices in our dataset. We compare the results obtained on the NVIDIA K20m **(A)**, the NVIDIA RTX 2060 **(B)**, and the NVIDIA RTX 3080 **(C)**. Although CSR is the fastest data structure in many cases, there is a noticeable number of cases where the other formats appear to be superior. The performance differences between the matrix formats are higher on the Tesla K20m **(A)** and the NVIDIA RTX 2060 **(B)** than on the RTX 3080 **(C)** especially for the ELLPACK-R matrix format. The orange line depicts the median, the green triangle the mean and the circle denote outliers.

Figure 5 depicts the average performance on the 3,000 data points in our dataset. The orange line represents the median of the obtained values and the green triangle represents the mean. The CSR format outperforms the other two formats in most cases on the K20m (Figure 5A) and the RTX 3080 (Figure 5C), as the average performance of ELLPACK-R and dense is lower than 1.0. However, there is a noticeable number of values >1.0, indicating that some matrices benefit from another format than CSR. We also found that the results on the RTX 2060 (Figure 5B) are different in the sense that the ELLPACK-R outperforms in many cases the CSR format which is represented by the average >1.0.

Comparing the results obtained on the three investigated CUDA devices supports the claim of Balaprakash et al. (2018). The performance behavior of a given implementation can drastically change with evolving hardware. The relative performance of our ELLPACK-R and dense implementations toward the CSR implementation indeed shrinks noticeably.

### 4.3. Automatic Format Selection

**Figure 6:**
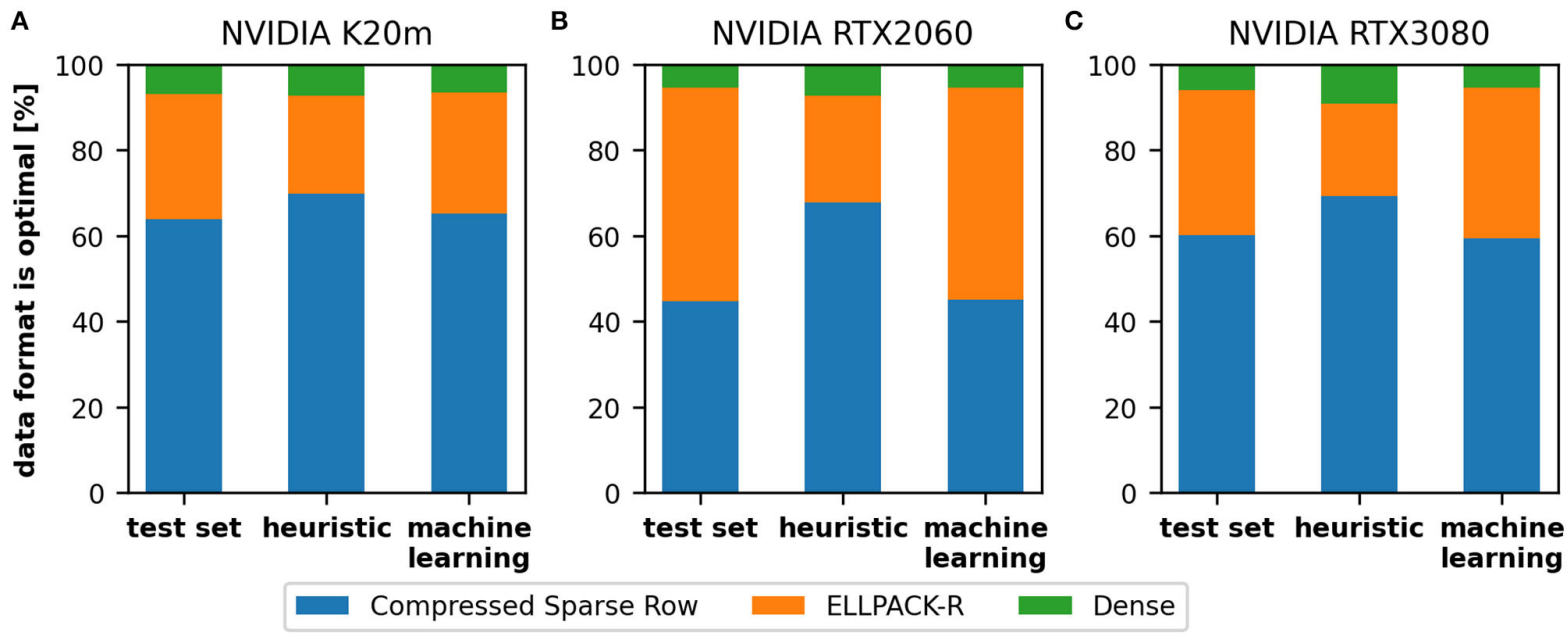
The distribution of selected formats on three GPUs: NVIDIA K20m **(A)**, NVIDIA RTX 2060 **(B)**, and NVIDIA RTX 3080 **(C)**. We compare the data (left), the heuristic (middle), and the machine learning model (right) for each GPU. We can see that our heuristic tends to select the compressed sparse row (blue bars) in too many cases, which leads to lower performance, in particular on the NVIDIA RTX 2060.

In this section, we report on the results of the two strategies for automatic format selection: the heuristic and the predictive machine learning approach. We compare the results on the K20m (Figure 6A), the RTX 2060 (Figure 6B), and the RTX 3080 (Figure 6C). Considering the distribution of the selected formats, we generally notice that there is no significant difference between the K20m and the RTX 3080 but the results of RTX 2060 appears to deviate. Furthermore, the machine learning model delivers more accurate results than the heuristic, especially on the RTX 2060. The heuristic tends to select on all three devices the CSR (blue bars) in too many cases, in particular on the RTX 2060. As noted earlier, this might be improved by device-specific thresholds used in the second stage of the heuristic. The machine learning model was able to select in 95.67% (K20m), 93.0% (RTX 2060), and 94.83% (RTX 3080) of the cases the correct format resulting in the fastest computation time. The selection of the heuristic was in 87.67% (K20m), 71.67% (RTX 2060), and 77.83% (RTX 3080) of the cases correct. We hypothesize that device-specific decision thresholds could improve the performance achieved on the RTX 2060 and RTX 3080, but it would be difficult to derive these thresholds on all possible hardware. It might be interesting to note that CSR format was in 63.83% (K20m), 44.67% (RTX 2060), and 60.17% (RTX 3080) of the cases the correct format.

### 4.4. Validation and Stability of the Machine Learning Approach

The performance of the ML approach depends on the correct selection of features and the size of the dataset dedicated to training and testing. However, the choice of a basic cross-validation method (random split of the data into 80% for training and 20% for testing) is not sufficient to estimate the appropriateness of the trained model, since it may have by coincidence excellent results only on the part selected for testing (20%). To avoid this issue, we have opted for the repetitive cross-validation method (Section 4.4.1). To define the proper size of the data required to obtain a stable model (a high accuracy with the lowest standard deviation), we also perform tests (using the repetitive cross-validation method) on different dataset sizes (Section 4.4.2).

#### 4.4.1. Cross-Validation

The five-fold cross-validation procedure divides the data set into five non-overlapping folds. During each iteration of the process, a fold is retained as a test set, while all others are used for the training. In the end, a total of five models are fitted and evaluated on the five retained test sets, and the average performance accuracy is calculated. This procedure is repeated ten times, and the mean performance across all folds and all repetitions is reported.

**Figure 7:**
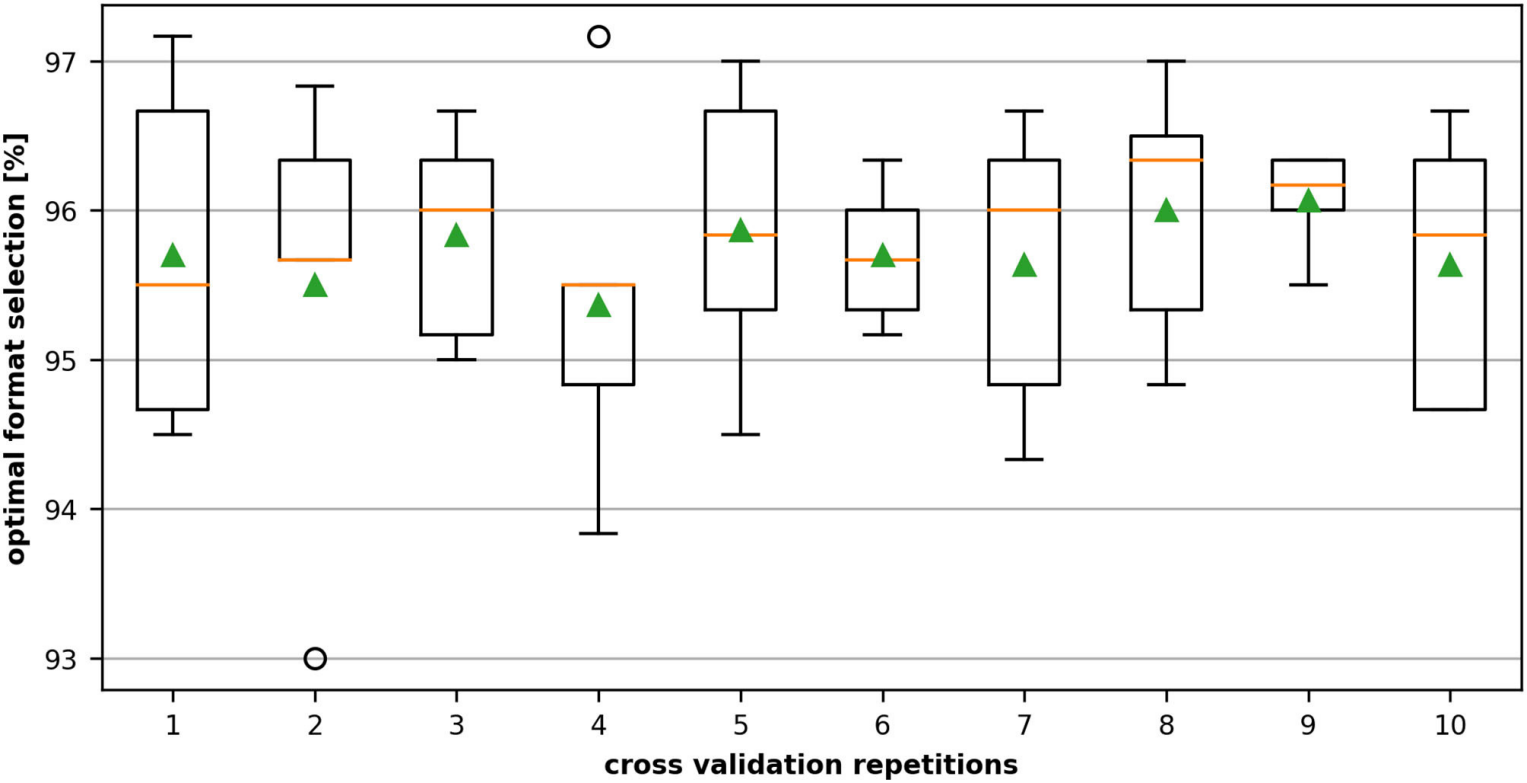
Ten repeated cross-validations on 3,000 data samples recorded on the NVIDIA Tesla K20m. The dataset is divided into five non-overlapping folds. During each validation stage, four folds containing 2,400 samples are used for training, and the remaining fold with 600 units is used for testing the accuracy of the best format selection. The middle (orange) line of the box is the median, the green triangle the mean and the circles denote outliers.

Figure 7 shows the variation of the performance of the 10 repetitive five-fold cross-validations applied on the dataset of the NVIDIA K20m. We can see that for the dataset with 3,000 data points, the optimal performance selection rate slightly varies depending on the fraction of data selected as training set but retains a high level of correctness over 93% and therefore still outperforms the heuristic.

#### 4.4.2. Influence of the Size of the Dataset

Generating the dataset can be quite time-consuming: the generation of the 3,000 data points required 2–3 days in this case. We therefore performed experiments (multiple repetitive five-fold cross-validations with varying each time the size of the dataset) to define the smallest dataset size enabling us to achieve a good accuracy of the selection of the correct matrix format. Bayesian optimization using Optuna for 150 trials is used to select the best architecture in each case.

**Figure 8:**
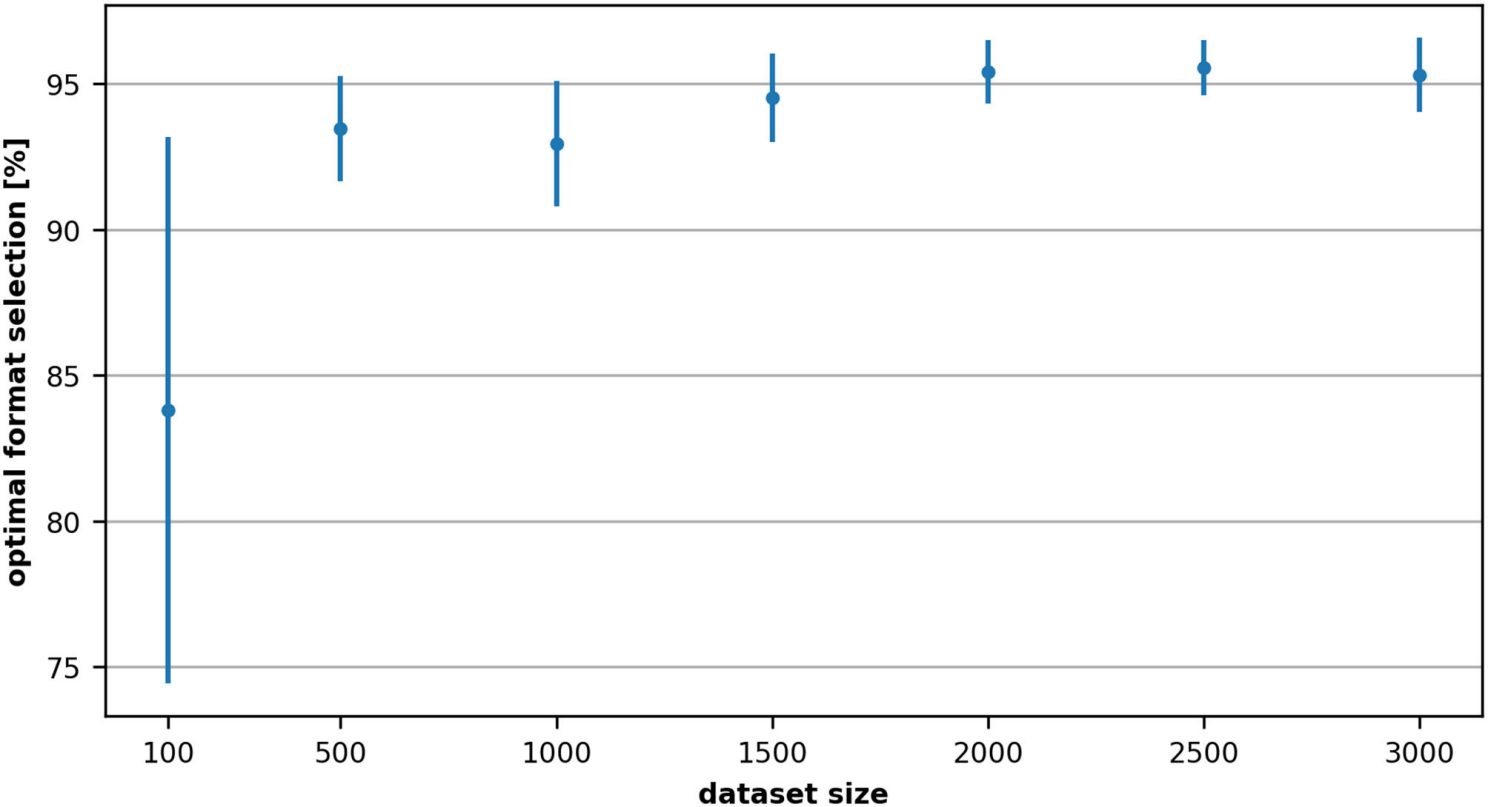
Variation of the accuracy of the optimal format selection with respect to the number of samples used for training (NVIDIA Tesla K20m). Each measurement and its corresponding standard deviation represents the average of 10 repeated cross-validations.

Figure 8 shows the accuracy variation of the optimal format selection with respect to the number of samples used for training. As one would expect, the performance increases with the size of the dataset. However, already with one-third of the dataset we could achieve an accuracy of 92.94% for the selection of the optimal format.

## 5. Discussion

Continuous transmission is a dominating computation kernel for rate-coded neural networks (Dinkelbach et al., 2012) that corresponds to the sparse matrix vector multiplication, a well-investigated topic by many researchers over decades on various hardware platforms. In this article, we investigated the application of the ELLPACK-R and dense format derived from the literature and study their performance within the neural simulation framework ANNarchy.

As stated in the literature, there is no “one-size-fits-all” solution, although the CSR format achieves a good performance in many cases, which was also shown, e.g., by Benatia et al. (2018) or Chen et al. (2019). Using a larger set of connection matrices, we have shown that the usage of different matrix formats can help to improve the performance on CPUs as well as GPUs by distinguishing between sparse and dense matrices (Section 4.1). For GPUs, we further studied the ELLPACK-R format proposed by Vázquez et al. (2011) in addition to our CSR implementation (Dinkelbach et al., 2012). In Section 4.2, we have shown that CSR is in many cases the best format, but it can be outperformed by a noticeable factor by the ELLPACK-R and the dense matrix format. In summary, the availability of different sparse matrix formats can be used to improve the performance but the selection is not trivial, as expected from the literature (e.g., Liu and Vinter, 2015a).

In the case of heavy simulations, a user-friendly simulation environment should measure and select the right sparse matrix format for a specific network. We presented a first automatic selection based on some simple rules which we derived from experiments and which is implemented in ANNarchy 4.7.1.1. We have also shown that this heuristic-based selection can be improved by the help of machine learning techniques. Our approach using machine learning techniques is comparable to the work of Lehnert et al. (2016) and Benatia et al. (2018). Based on a set of features, we build up a neural network which predicts the performance of the format. Lehnert et al. (2016) used computational time for the performance evaluation while we used GFLOPs as a metric. Both our work and that of Lehnert et al. (2016) uses regression for the prediction of the performance of the data format. Contrary to the previously discussed works, we do not use a fixed network but use the hyperparameter optimization framework Optuna to find a suitable network configuration for a given dataset. There is an important caveat: Comparing matrix formats using FLOPS as a metric generates a hardware dependency (Langr and Tvrdik, 2016), which we also observed in our recorded data (see Section 4.2). This means that the users need to generate the dataset on their own machine, which requires several hours up to a few days for the data generation, although the results in Section 4.4.2 suggest that the number of required data points can be reduced.

The present work demonstrates the performance improvements that can be reached by using the ELLPACK-R format in ANNarchy. However, the ELLPACK/ELLPACK-R formats require more memory caused by padding zeros for strongly varying row lengths and therefore, Bell and Garland (2009) proposed a Hybrid format, which combines an ELLPACK format for most entries, and those elements which are in the long rows are stored in a separate coordinate format. This was not the case in our dataset, and its not clear to us how relevant this is for neurocomputational models, as this would mean that the number of synapses per neuron vary strongly within one projection. The present CSR implementation could be further optimized for short rows using the CSR-stream implementation proposed by Greathouse and Daga (2014), although this introduces another hyper parameter: the number of nonzeros processed by one warp. The CSR5 storage format (Liu and Vinter, 2015a) introduces additional two hyperparameters but should be efficient for SIMD-capable CPUs, GPUs, or other accelerators like the Xeon Phi, while introducing a memory overhead around 2% of the original CSR (Liu and Vinter, 2015b).

Other works focus on the grouping of rows into computation blocks, i.e., by slicing the matrix into pieces, as done for the CSR (e.g., Oberhuber et al., 2011) or the ELLPACK format (e.g., Monakov et al., 2010; Kreutzer et al., 2014). Kreutzer et al. (2014) highlight that their modified sliced ELLPACK format is applicable to GPUs as well as SIMD-capable CPUs. Another class of formats proposed in the literature are blocked formats such as the blocked compressed sparse row (BSR or BCSR, e.g., Choi et al., 2010; Verschoor and Jalba, 2012; Eberhardt and Hoemmen, 2016; Benatia et al., 2018) or the blocked ELLPACK format (Choi et al., 2010). The idea is that matrix is split into several small dense matrices. As these sub-matrices are dense, a coalesced and fully cacheable access to the dense vector is possible, which is desirable for performance (Temam and Jalby, 1992; Im and Yelick, 2001; Im et al., 2004; Goumas et al., 2008; Williams et al., 2009). These formats appear to be efficient if the nonzeros in a matrix are clustered, although the selection of the correct block size can be challenging (Im and Yelick, 2001). For matrices where the nonzeros are widely spread, the memory overhead will be too large and no performance benefit can be expected in comparison to other formats.

The present work focuses on the performance prediction for sparse matrix formats on GPUs. Nonetheless, the same procedure can be applied for CPUs. Preliminary tests with the current ANNarchy 4.7.1 release has shown that the performance differences between formats are small in comparison to the differences observed on GPU. This hardens the correct performance prediction and opens the question of whether the approach is necessary at all. It is important to note that the recent implementations of our CPU formats are not comparable to highly optimized libraries like OSKI, SPARSITY, or ATLAS, as low-level optimization like padding, local store blocking or register blocking (e.g., presented in Im and Yelick, 2001; Im et al., 2004; Williams et al., 2009) are still missing. We started to apply such optimizations, e.g., hand-written SpMV which improve the performance (see Section 4.1), but this increases the complexity of the code generation noticeably. Nonetheless, we have implemented in the ANNarchy 4.7.1.1 the heuristic selection of dense matrices instead of sparse matrices.

Brian2 (Stimberg et al., 2019), GeNN (Yavuz et al., 2016) as well as ANNarchy do not switch the floating precision from double to single precision automatically. As highlighted by Hopkins et al. (2020), this could lead to numerical errors whose importance need to be evaluated by the modeler. However, the performance improvement on GPUs and CPUs (especially using SIMD extension) could be noticeable. The reduction of precision can improve the performance of the SpMV, e.g., shown by Bell and Garland (2009) or Greathouse and Daga (2014) and is therefore beneficial for the simulation of rate-coded models (Dinkelbach et al., 2012). Yavuz et al. (2016) have shown that the choice of single precision in context of two spiking models at different scales can improve the performance.

The presented findings may also be of interest for the implementation of spiking networks. The currently available spiking simulators use either CSR-like (e.g., Brian2, GeNN, coreNeuron; Kumbhar et al., 2019), dense (e.g., GeNN) or object-oriented (NEST) representation of synapses, while also using code generation approaches (see Blundell et al., 2018 for a recent review). At the very least, the differentiation between sparse and dense matrices could be helpful for some models as shown by Yavuz et al. (2016), as the usage of dense matrices does not break coalescence as CSR does (e.g., Dinkelbach et al., 2012; Yavuz et al., 2016). The computational load induced by the spike propagation can be quite low in comparison to the update of neural equations (Plesser and Diesmann, 2009), so there is a chance that the overhead induced by the sparse matrix format can have a negative impact on performance.

Ongoing work will target the application of other sparse matrix formats for the simulation of rate-coded and spiking models in ANNarchy. For rate-coded models, this could be formats which use structural properties, such as the diagonal format. Some neuro-computational models developed in our lab (e.g., Jamalian et al., 2017) contain matrices which have a banded matrix structure. A promising direction may be the implementation of sliced matrix formats (e.g., Kreutzer et al., 2014). For spiking models, the compressed sparse blocks format (CSB, Buluç et al., 2009, 2011) could be beneficial for the implementation of spiking models with plasticity rules. The CSB format is proposed to be suitable for the SpMV as well as the transposed SpMV, an uncommon property for SpMV formats (Buluç et al., 2009; Steinberger et al., 2016). With respect to the machine learning model, reducing the number of required data points is critical, as users will likely not be patient enough to gather the necessary data. Active learning methods (Cohn et al., 1996) may be used to allow the ML network to ask for additional samples where its uncertainty is maximal, focusing data generation to the most interesting regions.

## Data Availability Statement

The datasets presented in this study can be found in online repositories. The names of the repository/repositories and accession number(s) can be found at: Neural simulator ANNarchy: https://github.com/ANNarchy/ANNarchy (zenodo 10.5281/zenodo.6417924); Scripts for simulation/analysis: https://github.com/hamkerlab/Dinkelbach2022_ANNarchyAutoTuning (zenodo 10.5281/zenodo.6534573).

## Author Contributions

HD and B-EB designed and performed the research, programming, and data analysis. JV and FH guided the research. FH acquired the funding. HD writing first draft. HD, B-EB, JV, and FH writing, reviewing, and editing. All authors contributed to the article and approved the submitted version.

## Funding

This work was supported by the Deutsche Forschungsgemeinschaft (DFG) with the project Auto-tuning for neural simulations on different parallel hardware (DFG HA2630/9-1). The publication of this article was funded by the Deutsche Forschungsgemeinschaft (DFG, German Research Foundation) project number 491193532 and the Chemnitz University of Technology.

## Conflict of Interest

The authors declare that the research was conducted in the absence of any commercial or financial relationships that could be construed as a potential conflict of interest.

## Publisher's Note

All claims expressed in this article are solely those of the authors and do not necessarily represent those of their affiliated organizations, or those of the publisher, the editors and the reviewers. Any product that may be evaluated in this article, or claim that may be made by its manufacturer, is not guaranteed or endorsed by the publisher.

## Footnotes

^1^This means that the data of a column is stored continuously in memory instead of storing a row continuously (which is referred to as row-major).

^2^We use SIMD intrinsics which should not be confused with actual inline assembly (for more details, see: https://www.intel.com/content/www/us/en/docs/intrinsics-guide/index.html).

^3^Project homepage: http://math-atlas.sourceforge.net/.

^4^A warp is a group of 32 CUDA threads which process a given set of instructions at the same time. Even though they can proceed in the code concurrently, the efficiency rises if their execution does not diverge (Bell and Garland, 2009).

^5^ https://doi.org/10.5281/zenodo.5645375

^6^ https://doi.org/10.5281/zenodo.6417924

^7^The march flag let the compiler generate the code for a specific CPU architecture. Providing *native* let the compiler determine the CPU automatically. For more details, see https://gcc.gnu.org/onlinedocs/gcc/x86-Options.html.

^8^For more details, see https://gcc.gnu.org/onlinedocs/gcc/Optimize-Options.html.

^9^For more details, see https://gcc.gnu.org/wiki/FloatingPointMath.

^10^ https://docs.nvidia.com/cuda/cuda-compiler-driver-nvcc/index.html#ptxas-options-opt-level
