# Supplementary material for: Auto-Selection of an Optimal Sparse Matrix Format in the Neuro-Simulator ANNarchy
Source: Front Neuroinform. 2022 May 23;16:877945. doi: 10.3389/fninf.2022.877945 (PMC9169689; doi:10.3389/fninf.2022.877945)
Supplement: Supplementary file 1 [file Data_Sheet_1.PDF]

Supplementary Material

1 HARDWARE CHARACTERISTICS

The table below depicts the major hardware characteristics of the three devices used in the present article. We used the approach suggested by Mark Harris [1] to obtain these values using the *cudaDeviceProp* structure available in the CUDA runtime API [2]. Note that the number of CUDA cores is not available directly. One needs to multiply the *multiProcessorCount*, i.e. the number of available streaming multiprocessors (SMs) by the number of cores for one streaming multiprocessor for the architecture of the graphics card. Note that the core clock and especially the peak memory bandwidth are theoretical values.

GPU	Architecture	CUDA cores (SMs)	Core Clock	Peak Memory Bandwidth
NVIDIA K20m	Kepler	2496 (13)	706 MHz	208 GB / s
NVIDIA RTX 2060	Turing	1920 (30)	1820 MHz	336 GB / s
NVIDIA RTX 3080	Ampere	8704 (68)	1800 MHz	760 GB / s

2 MEMORY CONSUMPTION

As discussed in the article, the major idea of sparse matrix formats is to represent the nonzeros in a way that the sparse matrix vector multiplication can be performed most efficiently. For this purpose, some formats take a memory overhead into account. In this section, we make an analysis of the estimated memory consumption for the compressed sparse row (CSR, Eqn. S1), ELLPACK-R (Eqn. S2) and dense (Eqn. S3) formats where M is the number of rows, N the number of columns, nnz the number of nonzeros and max_{rl} the maximum row length in the matrix. We further have two data types *integer* for indices (4 bytes) and *double* for the nonzeros (8 bytes).

$$mem_{CSR} = (M + 1) \cdot 4 + nnz \cdot 4 + nnz \cdot 8 \quad \text{(S1)}$$

$$mem_{ELLR} = M \cdot 4 + M \cdot max_{rl} \cdot 4 + M \cdot max_{rl} \cdot 8 \quad \text{(S2)}$$

$$mem_{Dense} = M \cdot N \cdot 8 \quad \text{(S3)}$$

Using this equations, we can compute the memory consumption for a given format and then, by dividing with the number of nonzeros, we obtain the bytes required for a single nonzero. Figure S1 depicts this value as a function of the matrix density. We can see that the memory requirement is almost constant for the CSR (blue) format. The requirement of the ELLPACK-R (orange) can be high if the maximum row length (which determines the dense matrix size in this format) diverges largely from the average number of nonzeros. In this case, a large number of unnecessary elements are allocated in the memory. We can see the effect when comparing the CSR (Fig. S2, left) and the ELLPACK-R (Fig. S1, middle) memory consumption in bytes per nonzero. In the case where all rows are equally long, the consumption of CSR and ELLPACK-R is almost the same, as then $M \cdot max_{rl}$ is equal to nnz. We can further see that, for high densities (above 60-80%), the bytes per nonzero ratio for the dense (green) matrix gets below the required bytes per nonzero for the other sparse matrix formats.

[1] `https://developer.nvidia.com/blog/how-query-device-properties-and-handle-errors-cuda-cc/`

[2] `https://docs.nvidia.com/cuda/cuda-runtime-api/structcudaDeviceProp.html#structcudaDeviceProp`

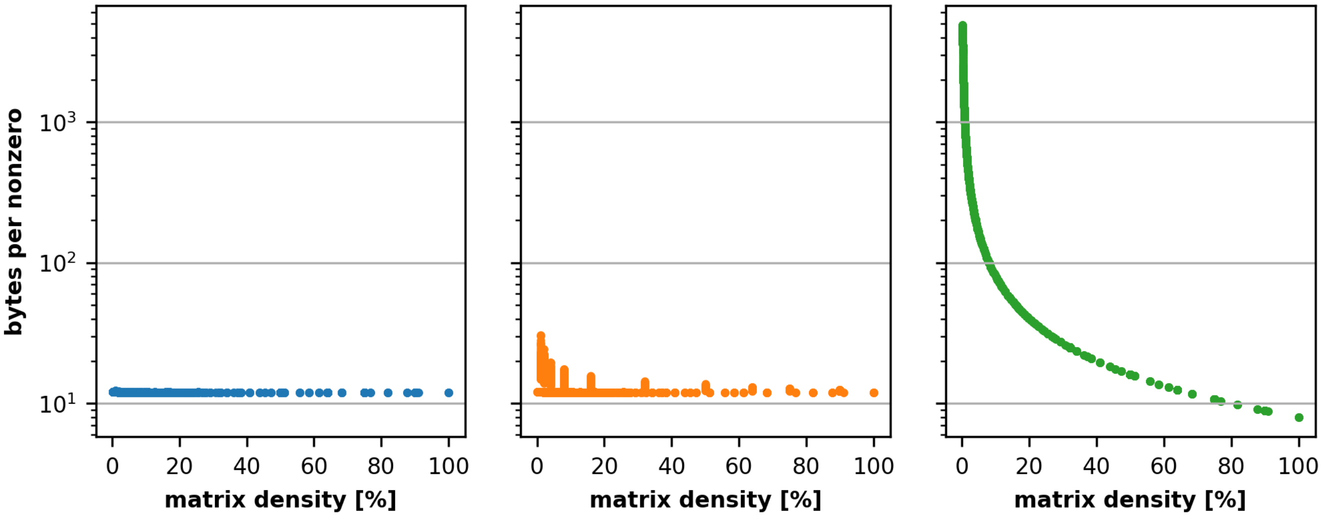

Figure S1. The figure depicts the required bytes necessary to represent a nonzero as a function of the matrix density. We compare the three formats CSR (blue), ELLPACK-R (orange) and dense (green).

3 DECISION BOUNDARY FOR THE HEURISTIC

In the heuristic presented in this article, we have two decision thresholds: One threshold for differentiation between dense and sparse formats and one for the differentiation between ELLPACK-R and compressed sparse row (CSR).

3.1 Dense vs. Sparse

Figure S2 shows the performance measured as GFLOPs as a function of the matrix density for the three graphic cards: NVIDIA K20m (top row), NVIDIA RTX 2060 (middle row) and the NVIDIA RTX 3080 (bottom row). The chosen threshold for selecting the dense matrix of 60% is chosen based on three observations: First, the performance of the CSR (left column, blue) seems to reach a saturation point. The ELLPACK-R (middle column, orange) performs in many cases worse than the CSR as well as dense (right column, green). For densities above 60%, the dense matrix performs best or at the level of the other two formats.

3.2 ELLPACK-R vs. Compressed Sparse Row

As pointed out in the article, one can derive from the implementation the hypothesis that the average number of nonzeros per row is the key factor to decide which format should be used. However, we need to determine the threshold value for this decision. We compute first the relative performance between ELLPACK-R and CSR. Figure S3 depicts the obtained ratio as a function of the average number of nonzeros per row. We then use the *scipy.optimize.curve_fit*[3] routine to fit an exponential function $y = a \cdot \exp(-b * x) + c$. The curve is fitted for each device individually and depicted as a red line. We then determine the point where this function crosses the 1.0 mark and identify 128 as threshold on the K20m (Fig. S3, A). We also noticed that this point seems to be applicable for RTX 3080 GPU (Fig. S3, C) while a larger block size might be suitable for the RTX 2060 (Fig. S3, B).

[3] https://docs.scipy.org/doc/scipy/reference/generated/scipy.optimize.curve_fit.html

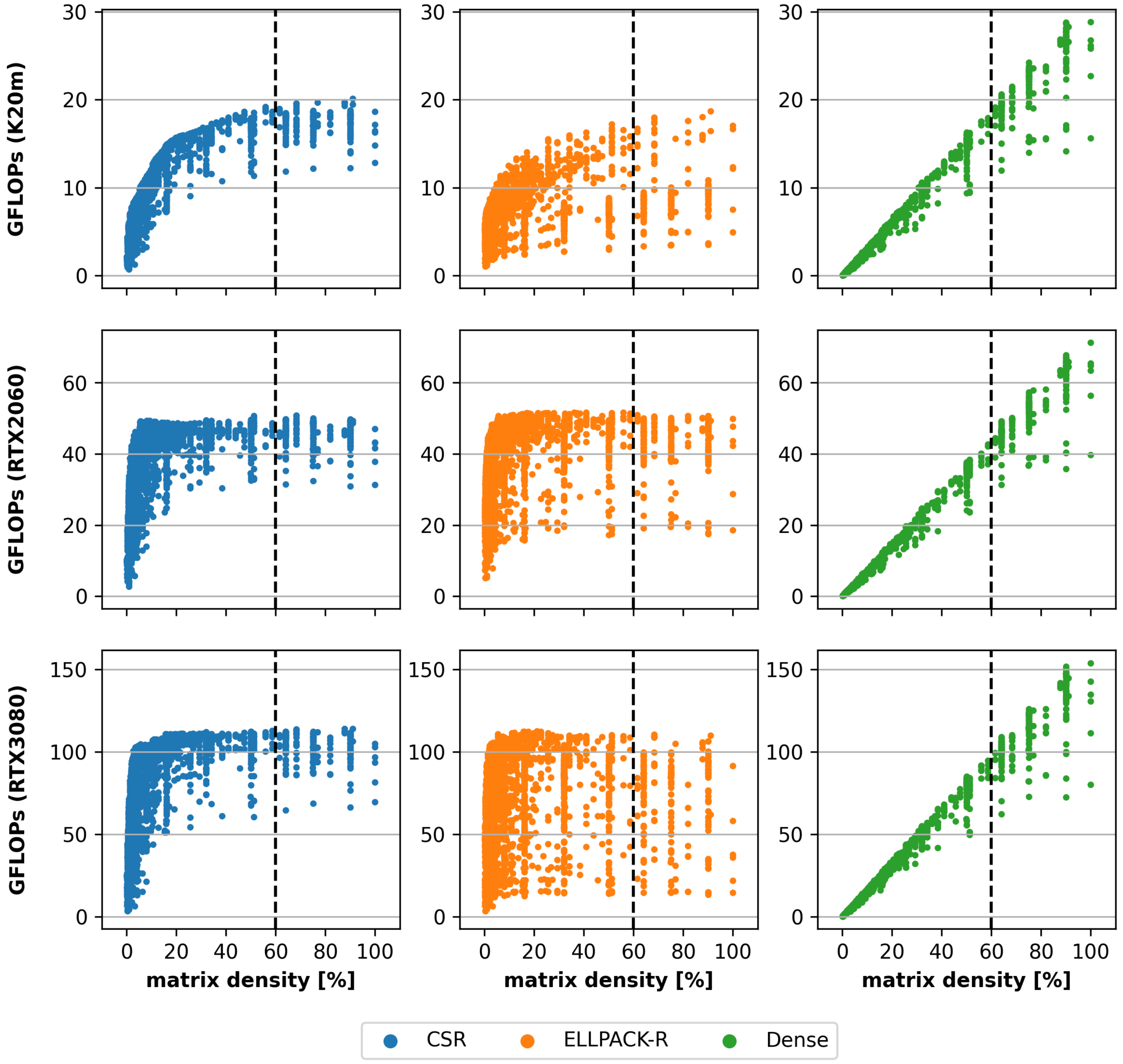

Figure S2. The figure depicts the achieved performance as a function of the matrix density for our three investigated formats CSR (blue), ELLPACK-R (orange) and dense (green). We measured the data on three different CUDA devices: NVIDIA K20m, NVIDIA RTX 2060 and NVIDIA RTX 3080. The dashed line indicates the chosen threshold of 60% density.

4 THE INFLUENCE OF THE *-FFAST-MATH* (CPU) / *–USE_FAST_MATH* (GPU) FLAG

As outlined in the introduction of the manuscript, the interaction of compiler flags with each other are hard to predict. Therefore, we want to reduce the number of compiler flags to a minimum. On the other side, a comparison should be performed with the best possible configuration. We want to demonstrate in this section the influence of the *-ffast-math* flag for g++ and the *-use_fast_math* flag for the nvcc (NVIDIA CUDA compiler). This should explain our choice of flags for the performed benchmarks. As described

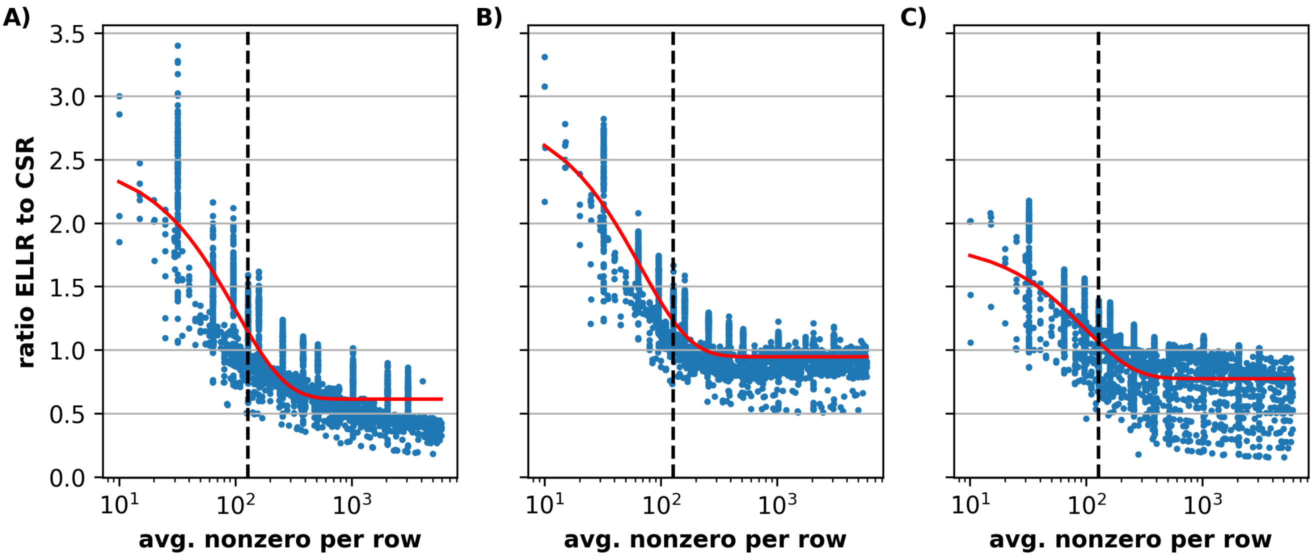

Figure S3. The figure depicts the relative performance of ELLPACK-R to CSR as a function of the average row length per row. We fitted a exponential function to the data point (red curve). The dashed line indicates the chosen threshold of 128 elements.

in section 4, we configured the g++ compiler with *-march=native -O3 -ffast-math* and the nvcc compiler without further flags as the nvcc compiler automatically enables a set of optimizations. It is worth noting that *-use_fast_math* is not part of the automatically enabled optimizations.

4.1 A brief introduction of the flags

According to the documentation[4], *-ffast-math* enables a set of optimizations for the g++ compiler. However, it is also mentioned that these flags may violate the IEEE compliance for floating operations. Therefore it is not enabled by default by any of the -O option aside of -Ofast[5]. This is particularly important for rounding or handling of not-a-numbers appearing in math operations, e.g. the division of two values. For nvcc, the usage of *–use_fast_math* changes rounding rules but it also forces the compiler to replace functions like pow() or exp() by their intrinsic counter part, which may violate the IEEE compliance (see the documentation[6] for more details). For the latter, it is assumed that it improves the performance but it also may reduce the accuracy. Therefore, the CUDA programming guide recommends that these transformations should be used by the developer when it improves the performance and it was checked that it does not impair the results.

4.2 Influence on GPU

In this section, we analyze the influence of the *–use_fast_math* flag on the benchmark as described in section 4.1 of the manuscript. We present in Figure S4 the data for the NVIDIA K20m, but we also observed the same behavior on the other devices. As shown by the ratio between disabled (A) and enabled (B) condition, we can conclude that this flag does not seem to have a significant influence on the obtained performance neither for CSR (blue line) nor the dense (orange) format. The small deviations from 1 can be

[4] https://gcc.gnu.org/wiki/FloatingPointMath

[5] see also: https://gcc.gnu.org/onlinedocs/gcc/Optimize-Options.html

[6] https://docs.nvidia.com/cuda/cuda-c-programming-guide/index.html#intrinsic-functions

linked to the fact that we compare the mean over 15 trials only. A detailed description of the experiment and the measurement can be found in section 4 of the manuscript.

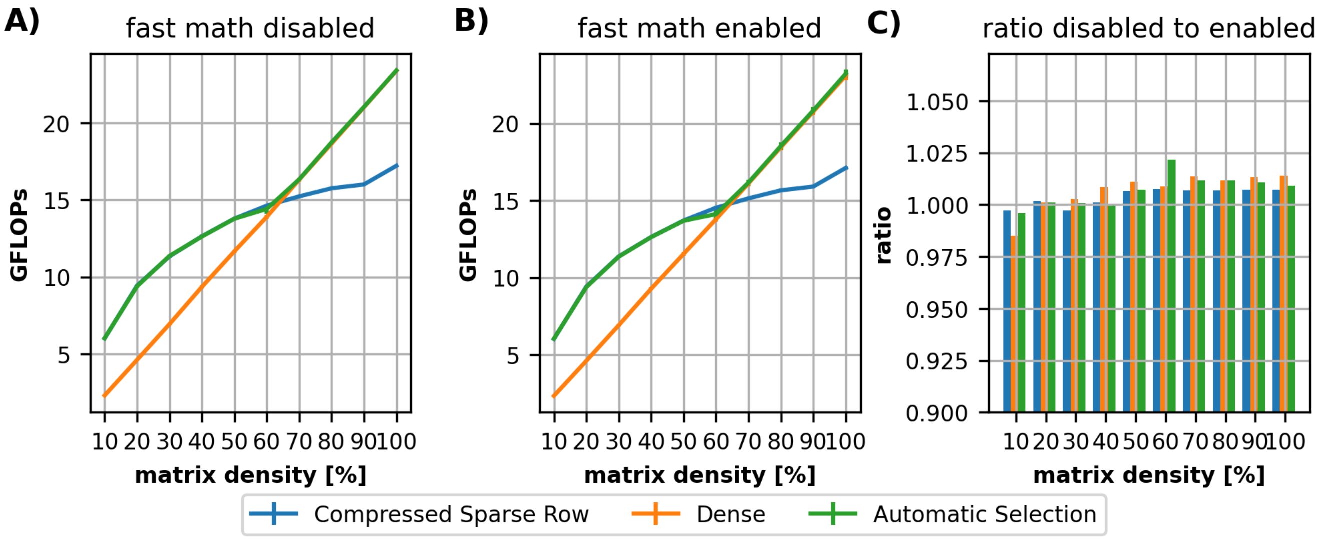

Figure S4. The figure analyzes the influence of the *-use_fast_math* flag on the experiment in section 4.1, when it's disabled (A) and enabled (B). For clarity of results, we provide the ratio between this two values in (C). For both formats CSR (blue) and dense (orange) we can not identify a significant difference.

4.3 Influence on CPU

While we observe no significant difference between the fast math optimizations being enabled or disabled on GPUs, we observe something different for CPUs. Figure S5 shows the performance obtained on the AMD Ryzen7 2700X CPU with disabled (A) and enabled (B) math optimizations. For the CSR format, both versions using no SIMD intrinsics (blue, straight line) and using SIMD intrinsics (blue, dashed line) do not suggest a significant influence of the *-ffast-math* flag. The same applies for the dense matrix using the SIMD intrinsics (orange, dashed line). The only difference is for the dense matrix implementation without SIMD intrinsics (orange, straight line). Using the vectorizer report (enabled by the *-fopt-info-vec*[7] flag) we could verify that an auto-vectorization is applied for the SpMV using the dense matrix format but not applied for the SpMV using CSR. As the code itself is not changed between enabled or disabled *-ffast-math* condition and only the dense result is affected, it appears that there is some interaction between the *-ffast-math* flag and auto-vectorization which is not clear to us at time of writing this manuscript.

[7] see for more details: `https://gcc.gnu.org/onlinedocs/gcc/Developer-Options.html`

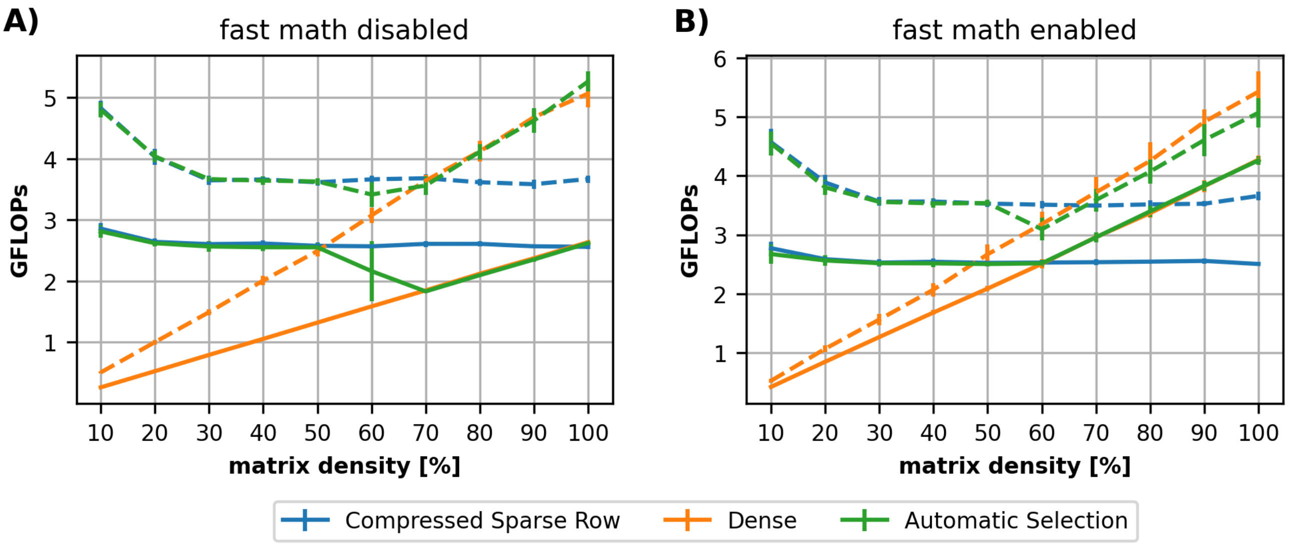

Figure S5. Results for the experiment in section 4.1 of the manuscript without (A) and using the -*ffast-math* flag (B). While there is no significant difference for the CSR format (blue, straight line), we observe a divergence between dense implementation without compiler intrinsics (orange, straight line). The implementation using intrinsics (dashed lines) are not affected in both formats.
